# Phosphorylation of Syntaxin‐1a by casein kinase 2α regulates pre‐synaptic vesicle exocytosis from the reserve pool

**DOI:** 10.1111/jnc.15161

**Published:** 2020-09-06

**Authors:** Vanilla (Hua) Shi, Tim J. Craig, Paul Bishop, Yasuko Nakamura, Dan Rocca, Kevin A. Wilkinson, Jeremy M. Henley

**Affiliations:** ^1^ School of Biochemistry Centre for Synaptic Plasticity Biomedical Sciences Building University of Bristol Bristol UK; ^2^ Department of Applied Sciences University of the West of England Bristol UK

**Keywords:** casein kinase 2α, phosphorylation, Syntaxin‐1, vesicle exocytosis

## Abstract

The t‐soluble NSF‐attachment protein receptor protein Syntaxin‐1a (Stx‐1a) is abundantly expressed at pre‐synaptic terminals where it plays a critical role in the exocytosis of neurotransmitter‐containing synaptic vesicles. Stx‐1a is phosphorylated by Casein kinase 2α (CK2α) at Ser14, which has been proposed to regulate the interaction of Stx‐1a and Munc‐18 to control of synaptic vesicle priming. However, the role of CK2α in synaptic vesicle dynamics remains unclear. Here, we show that CK2α over‐expression reduces evoked synaptic vesicle release. Furthermore, shRNA‐mediated knockdown of CK2α in primary hippocampal neurons strongly enhanced vesicle exocytosis from the reserve pool, with no effect on the readily releasable pool of primed vesicles. In neurons in which endogenous Stx‐1a was knocked down and replaced with a CK2α phosphorylation‐deficient mutant, Stx‐1a(D17A), vesicle exocytosis was also increased. These results reveal a previously unsuspected role of CK2α phosphorylation in specifically regulating the reserve synaptic vesicle pool, without changing the kinetics of release from the readily releasable pool.

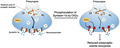

## Graphical abstract

Syntaxin‐1a (Stx‐1a) is an essential component of the pre‐synaptic release machinery. Stx‐1a is phosphorylated by the kinase CK2α, however, the consequent impact on pre‐synaptic exocytosis is poorly understood. We show that reducing Stx‐1a phosphorylation by knockdown of CK2α enhances pre‐synaptic vesicle exocytosis from the reserve pool, whereas over‐expressing CK2α reduces exocytosis. Replacement of endogenous Stx‐1a with a mutant that cannot be phosphorylated by CK2α enhances exocytosis from the reserve pool. Together, our results support a model whereby phosphorylation of Stx‐1a by CK2α acts to specifically restrict the size of the reserve pool of vesicles, resulting in reduced vesicle exocytosis.

**Abbreviations**
APs: action potentials
ARRIVE: Animal research: Reporting in vivo experiments
BGMSC: Biological and Genetic Modification Safety Committee
BSA: bovine serum albumin
CK1α: Casein kinase 1α
CK2α: Caesin kinase 2α
DIV: days in vitro
HRP: horseradish peroxidase
KD: knockdown
PBS‐T: phosphate‐buffered saline containing tween
ROI: region of interest
RP: reserve pool
RRID: Research Resource Identifier
RRP: readily‐releasable pool
SCR: scrambled
*SD*: standard deviation
SDS–PAGE: sodium dodecyl–sulphate polyacrylamide gel electrophoresis
*SEM*: standard error or the mean
shRNA: short‐hairpin RNA
SNARE: soluble NSF‐attachment protein receptor
Stx‐1a: Syntaxin‐1a
SypHy: synaptophysin‐pHluorin
WB: Western blot
WT: wild‐type

## 1 INTRODUCTION

A complex and highly orchestrated protein machine controls the calcium‐dependent fusion of synaptic vesicles with the pre‐synaptic membrane to mediate neurotransmitter release (Sudhof & Rothman, 2009). These vesicles exist in several functionally distinct pools, although the precise definition and regulation of the size of these pools is not fully elucidated. Briefly, following an initial release event from the readily releasable pool (RRP) of synaptic vesicles, which are immediately available for Ca^2+^‐triggered release (Rosenmund & Stevens, 1996), a slower, more sustained period of release can be mediated by mobilization of vesicles from a reserve pool that enables neurotransmitter release to be maintained during intense periods of synaptic activity (Rizzoli & Betz, 2005).

The vesicular docking and release of neurotransmitter requires fusion of the synaptic vesicle and pre‐synaptic membranes, a process that critically depends on the assembly of the soluble NSF‐attachment protein receptor (SNARE) complex (Sudhof & Rothman, 2009). A core component of the SNARE complex at the pre‐synaptic membrane is Syntaxin‐1 (Stx‐1). Stx‐1 contains a single C‐terminal transmembrane domain, a SNARE domain (known as H3) and an N‐terminal regulatory domain that binds Munc‐18‐1 (Padmanabhan et al., 2019; Rizo, 2018).

Stx‐1 exists in functionally distinct ‘open’ and ‘closed’ conformations (Dulubova et al., 1999). Munc18‐1 binds to Stx‐1a via two different modes. In mode 1, Munc18‐1 binding to the ‘closed’ conformation of Stx‐1a inhibits SNARE complex formation and therefore neurotransmitter release. In mode 2, Munc18‐1 interacts with a conserved N‐terminal domain of Stx‐1a and this interaction is thought to be critical in SNARE complex formation and neurotransmitter release. The mode 2 interaction is regulated by the action of another protein, Munc13, which catalyses the transition of Stx‐1a from ‘closed’ to ‘open’ conformations and is essential for vesicle priming (Rizo & Xu, 2015). Additionally, the mode 2 interaction is inhibited by phosphorylation of Stx‐1a at Ser14 (Rickman & Duncan, 2010). The combination of these two modulatory mechanisms allows precise control of SNARE complex formation, consequent synaptic vesicle fusion with the pre‐synaptic membrane, and neurotransmitter release (Sudhof, 2014).

Phosphorylation of Ser14 in Stx‐1a is catalysed specifically by the serine/threonine kinase Casein kinase 2α (CK2α) (Risinger & Bennett, 1999), which is ubiquitously expressed in all eukaryotic cells (Litchfield, 2003) but is particularly abundant in brain (Rebholz, Zhou, Nairn, Greengard, & Flajolet, 2013). CK2α has a wide range of functions including an essential role in maintaining pre‐synaptic stability in neurons (Bulat, Rast, & Pielage, 2014). However, little is known about the physiological role of Stx‐1a phosphorylation by CK2α in the regulation of release from the different synaptic vesicle pools.

Here we investigated the role of CK2α phosphorylation of Stx‐1a in neurotransmitter release from primary rat hippocampal neuronal cultures. We show that reducing CK2α‐dependent phosphorylation of Stx‐1a leads to an increase in sustained synaptic vesicle exocytosis. These data suggest that, in addition to involvement in the priming of synaptic vesicles, CK2a phosphorylation of Stx‐1a also regulates the apparent number of releasable synaptic vesicles.

## 2 MATERIALS AND METHODS

The experimental design of this study was not pre‐registered.

### 2.1 Plasmid constructs

To knock down expression of endogenous proteins, short‐hairpin RNAs (shRNAs) targeting casein kinase 1α (CK1α) (target sequence GCGTCACTGTAATAAGTTATT), CK2α (target sequence GCAATTGTACCAGACGTTAAC) or a non‐targeting control (target sequence AACGTACGCGGAATACTTCGA) were cloned into the plasmid pSUPER‐neo‐mCherry using BglII and XhoI. For lentiviral knockdown, the HI promoter‐shRNA cassette was PCR amplified and cloned into the PacI and XhoI sites of the lentiviral vector pXLG3‐PX‐GFP‐WPRE (Rocca, Wilkinson, & Henley, 2017). For over‐expression of CK2α, cDNA encoding rat CK2α was amplified by PCR and cloned into the KpnI and XhoI sites of pmCherry‐C3. As a control for over‐expression, empty pmCherry‐C3 was used. For Stx‐1a knockdown‐rescue, a dual expression pFIV vector expressing both an shRNA targeting rat Stx‐1a (target sequence CAGAGGCAGCTGGAGATCA; (Zhou et al., 2013)) and an shRNA‐resistant Stx‐1a‐IRES‐mCherry rescue cassette was used, as described and validated previously (Craig, Anderson, Evans, Girach, & Henley, 2015). The D17A‐Syt‐1a was produced by site‐directed mutagenesis. For synaptophysin‐pHluorin (SypHy) assays, synaptophysin‐pHluorin was expressed from the plasmid pcDNA3 (a gift from Ruud Toonen, Vrije Universiteit Amsterdam, Netherlands). The fidelity of all DNA constructs was confirmed by DNA sequencing (Eurofins Genomics).

### 2.2 Lentivirus production

Lentivirus was produced in HEK293T cells (ECACC, Research Resource Identifier, RRID:CVCL_0063), as described previously (Rocca et al., 2017), by co‐transfection of pXLG‐based lentiviral vectors with the helper vectors p8.91 (Addgene, Cat# 12263) and pMD2.G (Addgene, Cat# 12259). Forty‐eight hours later, lentivirus‐containing culture media was harvested, 0.45 µm filtered, and applied to cultured neurons.

### 2.3 Primary neuronal cultures

E18 pups harvested from pregnant female Wistar rats were sacrificed following UK Home Office Schedule 1 regulations via administration of an overdose of gaseous isoflurane anaesthesia to ensure complete unconsciousness followed by cervical dislocation. Pregnant females were housed alone with free access to food and water. Primary cultures are shared between group members to minimize animal use and neurons cultured from E18 pup litters from at least 20 independent dissections (i.e. 20 pregnant female rats) were used to generate the data presented. All animal care and procedures were carried out in full compliance with University of Bristol and ARRIVE guidelines, and the U.K. Animals Scientific Procedures Act, 1986. In addition, all experimental protocols were approved by University of Bristol Animal Welfare and Ethics Review Body (ethics approval number UIN: UB/18/004) panel and the Biological and Genetic Modification Safety Committee (BGMSC).

Dissociated hippocampal and cortical neuronal cultures were prepared as described previously (Carmichael, Wilkinson, Craig, Ashby, & Henley, 2018; Martin & Henley, 2004). Briefly, following dissection, isolated cortices and hippocampi were dissociated with trypsin and plated onto PLL (Sigma Aldrich, Cat# P2636)‐coated cell culture dishes or coverslips. Cells were plated into Neurobasal media (Gibco, Cat# 21103049) supplemented with 5% horse serum (Sigma Aldrich, Cat# H1270), B27 (1×, Gibco, Cat# A3582801), P/S (Gibco, Cat# 15140122) and 5 mM Glutamax (Gibco, Cat# 35050038) for 24 hr in a 37°C, 5% CO_2_ cell culture incubator. This media was then replaced with feeding media (as above but without horse serum and with 2 mM Glutamax). Neurons were plated at a density of 500,000 per 35 mm well for biochemistry and 200,000 per coverslip for imaging and used for experiments at the ages indicated.

Cortical neurons were used for validation of knockdown and knockdown‐rescue viral constructs because far more cells are generated per dissection and large numbers are required for biochemical experiments and western blotting. Hippocampal neurons were used for exocytosis assays because they are the most extensively used model in the literature for studying synaptic function.

### 2.4 Sodium dodecyl–sulphate polyacrylamide gel electrophoresis and western blotting

Proteins were separated by 8%–15% sodium dodecyl–sulphate polyacrylamide gel electrophoresis and resolved proteins were transferred from the gel onto 0.45 µm PVDF for western blotting. Following transfer, membranes were washed in phosphate‐buffered saline containing tween (PBS‐T) (8 mM Na_2_HPO_4_, 150 mM NaCl, 2 mM KH_2_PO_4_, 3 mM KCl, 0.1% (v/v) Tween‐20, pH 7.4) and blocked for 1 hr in 5% (w/v) bovine serum albumin. Membranes were incubated with primary antibody for 1 hr and were subsequently washed three times with PBS‐T followed by 50 min incubation in Horseradish peroxidase‐conjugated secondary antibody (Sigma Aldrich, Cat# A3682 (mouse), Cat# A5795 (rabbit); 1:10,000).

Primary antibodies were diluted in 5% (w/v) bovine serum albumin in PBS‐T for western blotting. The antibodies used were as follows: CK1α (Abcam, Cat# ab108296, RRID:AB_10864123) 1:2000; CK2α (Cell Signaling Technology, Cat# 2656, RRID:AB_2236816) 1:1,000; Syntaxin‐1 (Sigma Aldrich, Cat# SAB4200841) 1:15,000; phospho‐Syntaxin‐1a (Ser14) (Sigma Aldrich, Cat# SAB4504321) 1:2000; GAPDH (Abcam, Cat# ab8245, RRID:AB_2107448) 1:10,000.

Western blots were imaged using X‐ray films in a dark room using developer and fixer solutions. The blots were then scanned and quantified using FIJI (ImageJ studio). For western blot studies, *N* numbers refer to the number of neuronal cultures from independent dissections that were assayed.

### 2.5 Transfection of hippocampal neurons

Rat hippocampal neurons plated on 25 mm coverslips were typically transfected using Lipofectamine^®^ 2000 transfection reagent (Thermo‐Fisher, Cat# 11668019). For a 1 µg DNA transfection reaction, 1.5 µl of Lipofectamine^®^ 2000 was added to 100 µl of Neurobasal^®^ media, gently vortexed and left at room temperature (20°C) for 5 min. In a separate reaction, 1 µg of DNA was added to 100 µl Neurobasal^®^ media which was gently vortexed and added to the Lipofectamine^®^ 2000 containing mixture and incubated at room temperature (20°C) for 25 min. Coverslips were rinsed in Neurobasal^®^ media and then transferred to a 6‐well plate containing 1 ml of transfection media (Neurobasal^®^ media, 10% B27 and 10% glutamax) per well. The transfection mix was added dropwise to neurons and cells were incubated at 37°C for 2 hr. Coverslips were then rinsed in Neurobasal^®^ media before being transferred to the original conditioned media and were left for 4 days before being used in the Synaptophysin‐pHluorin exocytosis assay (Girach, Craig, Rocca, & Henley, 2013).

### 2.6 Synaptophysin‐pHluorin exocytosis assay

Primary hippocampal neurons prepared on coverslips were co‐transfected at days in vitro (DIV10) with pcDNA3‐SypHy and either (a) CK1α, CK2α or scrambled versions of shRNAs carried in a pSuper‐neo‐mCherry vector, (b) mCherry‐CK2α over‐expressing construct (or mCherry alone as a control), or (c) Syntaxin‐1a‐IRES‐mCherry knock‐down (KD) rescue constructs. The SypHy functional assay was performed when cells reached DIV14. Since constructs (a) to (c) all express mCherry as a fluorescent marker, only cells expressing both SypHy and mCherry were imaged.

SypHy experiments were performed as follows: post co‐transfection, neurons were mounted in a Warner stimulation chamber (Harvard Apparatus) connected to a Digitimer Constant Voltage stimulator and a Master‐8 pulse train generator (Burrone, Li, & Murthy, 2006; Girach et al., 2013). Exocytosis was triggered using electrical field stimulation, using 1 ms pulses (APs) of 50 V. Baseline recordings were taken using 2 Hz imaging for 30 s before a stimulation of 40 APs at 20 Hz was applied to release RRP; 10 s later a stimulation of 900 APs that last for 45 s at 20 Hz was applied so that reserve pool release, or bulk exocytosis, could be recorded. The electrical field stimulation experimental set up was arranged and optimized for use with the Nikon Eclipse Ti‐E C1 plus confocal microscope. Time‐lapse imaging was performed so that 512 × 512 pixel images were acquired using a 60× oil lens objective with medium pinhole and minimal pixel dwell time. All experiments were performed in HBS (25 mM HEPES, 140 mM NaCl, 5 mM KCl, 1.8 mM CaCl2, 0.8 mM MgCl2, 5 mM glucose, pH 7.4) with 50 µM D‐AP5 (Tocris, Cat# 0106), 25 µM CNQX (Tocris, Cat# 0190). Perfusion with NH_4_Cl (pH 7.4), was used to reveal total levels of Synaptophysin‐pHluorin loading of neurons at the end of each experiment. Images were taken at 2 Hz, using a CCD camera.

For each cell, 15 region of interests (ROIs) were analysed, with non‐responsive ROIs discounted. Parameters were also set to avoid saturation of signal and ROIs with saturated signal at the end point of the 3 min programme were excluded from data analysis. Data were first normalized to background levels (i.e. ΔF/F_0_), and then expressed as a percentage of fluorescence after NH_4_Cl perfusion (*F*_max_). All analysis for time‐lapse imaging was carried out using MacBiophotonics Image J software (NIH).

### 2.7 Stastical analysis, blinding and sample size calculations

Data were processed in Microsoft Excel and statistical analyses were carried out using Graphpad Prism^®^ (version 7.0). Prior to statistical analysis data were tested for normality using the Shapiro–Wilk normality test and statistical tests chosen appropriately. No outliers were removed prior to statistical analysis, however, for SypHy imaging, ROIs that did not respond, or were saturated at the end point of the experient were excluded from analysis. For statistical analysis on data that passed the normality test, *t* tests were used when comparing two groups. Where one of these groups was set to a predetermined value and lacked variance (e.g. when a control group was set to 100%), a one‐sample *t* test was used to determine whether the test group was significantly different from a set value (100% in this example). Where both groups exhibited variance, a Student's *t* test was performed. For comparing more than two groups, one‐way ANOVA was used, followed by Tukey's *post hoc* test to test for differences between groups. For data that did not pass the normality test, Mann–Whitney test was used for comparison of two groups, and Kruksal–Wallis followed by Dunn's multiple compairsons test for comparisons between three groups. In all cases, *p* < .05 was considered statistically significant.

For SypHy assays, slope analysis of endocytosis was performed by linear regression of the linear phase and decay rates and tau values derived from the equation of the curve. RRP analysis was performed by measuring peak RRP exocytosis by taking an average signal 2–3 s after stimulation of RRP release. Reserve pool (RP) exocytosis was measured by taking the area under the curve at the timepoints indicated, which encompassed the time of peak exocytosis. The time window used for RP analysis differed slightly between sets of experiments because of small differences in the time to peak RP exocytosis.

Plotted curves are presented as the mean ± *SEM*, whereas quantified data are presented as the mean ± *SD*. *N* numbers for biochemistry refer to the number of independent cell culture preparations assayed (i.e. from different dissections), whereas for imaging refer to the number of cells assayed per condition, from at least three independent cell culture preparations. No explicit blinding or sample size calculations were performed.

## 3 RESULTS

### 3.1 CK2α knockdown reduces phosphorylation of Ser14 in Stx‐1a

**Figure 1:**
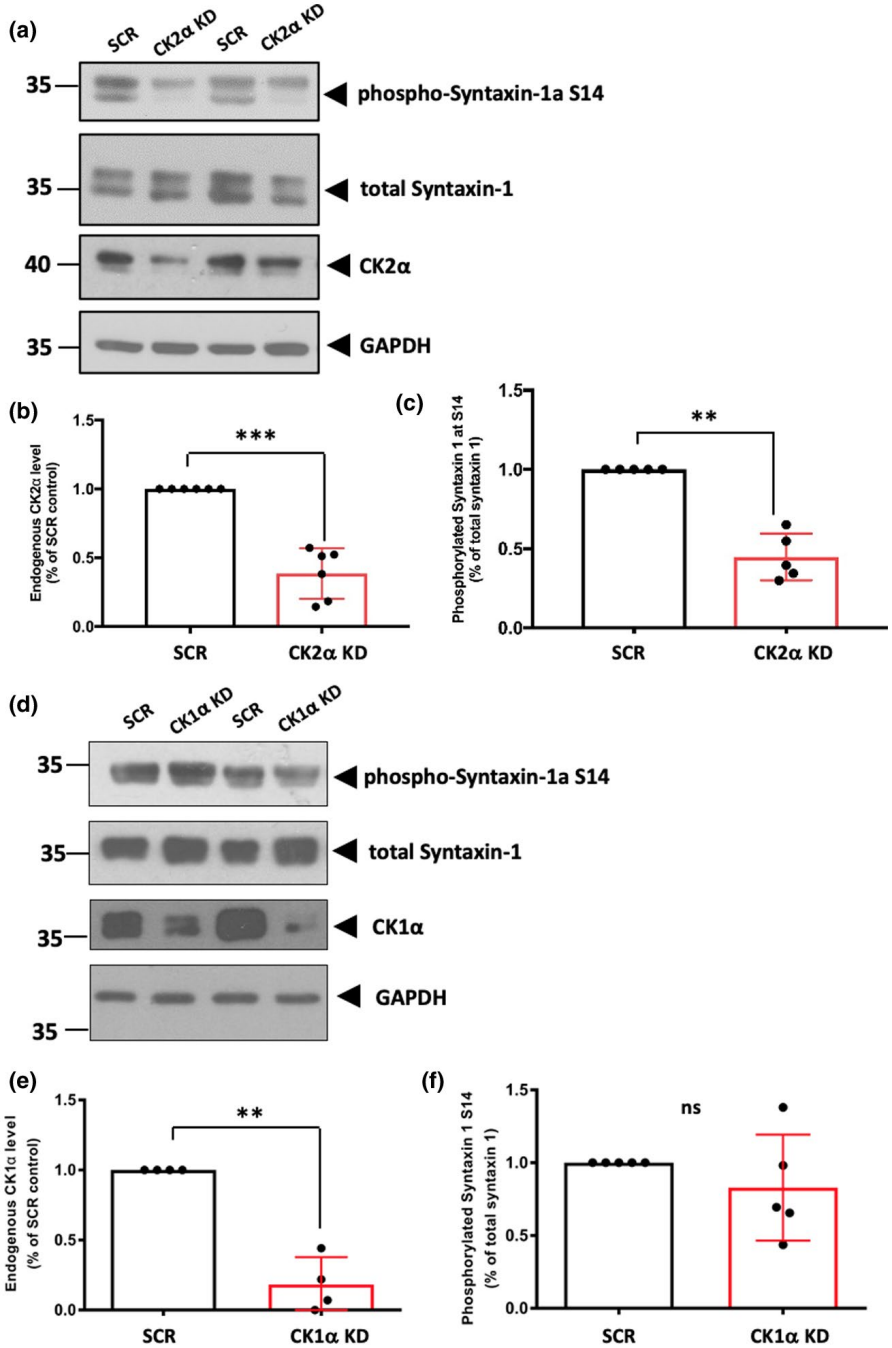
Phosphorylation of endogenous Syntaxin‐1a at S14 is selectively reduced by knocking down Casein kinase 2α (CK2α). (a, d) Representative western blots of Ser14 phosphorylated Syntaxin‐1a, total Syntaxin‐1a and CK2α (a) or Casein kinase 1α (CK1α) (d). DIV8 cortical neurons were infected with lentiviruses expressing a CK1α short‐hairpin RNA (shRNA), CK2α shRNA or a scrambled, non‐targeting shRNA for 6–7 days before being lysed and western blotted for both endogenous CK1α/CK2α and phospho‐Syntaxin‐1a (Ser14). GAPDH was used as a loading control. (b, e) Quantification of the efficiency of knockdown of endogenous CK2α (~60%; b) or CK1α (~80%; e) by their cognate shRNAs. One‐sample *t* test, **p < .01, ***p < .001. *N* = 6 independent cell culture preparations for CK2α and *N* = 4 for CK1α. Graphs show mean ± *SD*. (c, f) The proportion of phospho‐Syntaxin‐1a (Ser14) significantly decreased after knocking down endogenous CK2α (one‐sample *t* test, **p < .01), but it did not change after knocking down endogenous CK1α (one‐sample *t* test, *p* > .05). For analysis, phospho‐Syntaxin‐1a (Ser14) was normalized to total endogenous Syntaxin‐1. Both the higher and lower molecular weight bands that appeared on the blots were analysed together. Graphs show mean ± *SD*. *N* = 5 independent cell culture preparations samples

To investigate the involvement CK2α phosphorylation of Stx‐1a in pre‐synaptic vesicle release, we used lentiviruses encoding either a scrambled, non‐targeting shRNA, or shRNAs targeting CK2α or CK1α. The efficacy of KD was validated in cortical neurons by western blotting. Neurons were infected with lentivirus at DIV8, and lysed 6–7 days later. The CK1α shRNA reduced CK1α protein levels by ~80% and the CK2α shRNA reduced CK2α protein levels by ~60% compared to scrambled shRNA controls (Figure 1a,bd,e—example full blots shown in Figure S3).

Knockdown of CK2α, but not CK1α, resulted in a significant decrease in Ser14 phosphorylation of endogenous Stx‐1a (Figure 1c,f), as determined by western blotting with a phospho‐Stx‐1a (Ser14)‐specific antibody. There were no changes in levels of total Stx‐1a, confirming that Ser14 is specifically phosphorylated by CK2α. The immunoblots probed with the phospho‐Stx‐1a antibody detect a doublet, suggesting that more than one phosphorylated isoform of Stx‐1 is present in rat neurons. Two isoforms of Stx‐1, Stx‐1a and Stx‐1b, have identical amino acid sequences around Ser14 (Foletti, Lin, Finley, & Scheller, 2000). Both isoforms are expressed in the rat central nervous system (Inoue, Obata, & Akagawa, 1992; Kushima, Fujiwara, Sanada, & Akagawa, 1997), but possess different regional distributions (Ruiz‐Montasell et al., 1996). We note that knockdown of endogenous CK2α only significantly reduced of the lower band, which represents Stx‐1a, at Ser14.

### 3.2 CK2α knockdown enhances pre‐synaptic vesicle release

**Figure 2:**
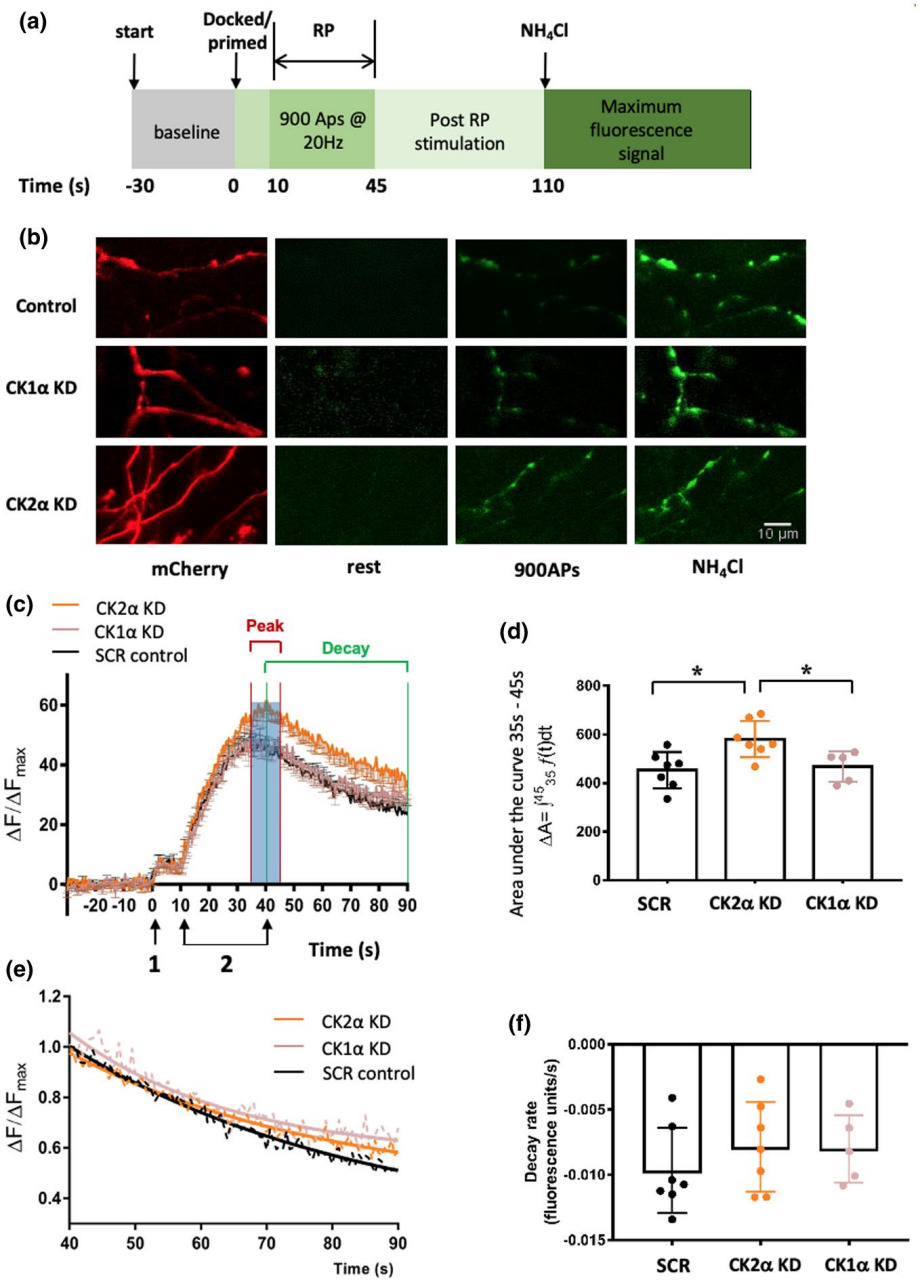
Casein kinase 2α (CK2α) knock‐down enhances pre‐synaptic vesicle release. (a) Schematic of experimental timeline and protocol. The baseline fluorescence was measured for 30 s at 10 frames per second. A brief stimulation of 40 action potentials (APs) @ 20Hz was then applied to release the readily releasable pool (RRP) of vesicles. The cells were then stimulated 10 s later with 900 APs @ 20Hz to activate the reserve pool (RP). The neurons were then monitored and after 110 s ammonium chloride was added to transiently collapse all intracellular pH gradients to pH 7.4 and provide a measure of maximum synaptophysin‐pHluorin (SypHy) fluorescence. (b) Representative images of neurons transfected with constructs expressing SypHy and the indicated short‐hairpin RNAs (shRNAs) (mCherry). Illustrative images of SypHy fluorescence prior to stimulation [rest, 0 s in (a)], after a stimulation at 900 APs at 20Hz [900 AP; 40 s in (a)] and after the NH_4_Cl wash to reveal maximal SypHy fluorescence. Scale bar = 10µm. (c) Exocytosis profile measured by SypHy signal in neurons transfected with a scrambled shRNA control (SCR; *N* = 7), Casein kinase 1α (CK1α) shRNA (CK1α knockdown, KD; *N* = 5) or CK2α shRNA (CK2α KD; *N* = 7). Stimulation 1, 40 APs @ 20Hz to release the RRP. Stimulation 2, 900 Aps @ 20Hz to release the entire recycling pool. Fluorescence normalized to baseline and expressed as a percentage of total SypHy signal obtained by an NH_4_Cl wash (pH 7.4). Graph shows mean ± *SEM*. (d) Quantification of peak areas under the curves from 35 to 45 s. One‐way ANOVA followed by Tukey's *post hoc* multi‐comparison, *p < .05. Data are presented as mean ± *SD*. SCR *N* = 7 cells from at least three independent cell culture preparations; CK1α KD *N* = 5; CK2α KD *N* = 7. (e) SypHy fluorescence decay profiles between 40 and 90 s normalized to the peak evoked signal. Decay profiles are all best described by exponential functions, and are presented as mean only without error bars for clarity. (f) Decay curves from (e) were calculated and quantified. *p* > .05 (One‐way ANOVA followed by Tukey's *post hoc* multi‐comparison), data are presented as mean ± *SD*. SCR *N* = 7 cells from at least three independent cell culture preparations; CK1α KD *N* = 5; CK2α KD *N* = 7

We next tested if CK phosphorylation altered vesicular release using the Synaptophysin‐pHluorin (SypHy) exocytosis assay. Briefly, the SypHy assay employs a pH‐sensitive fluorophore reporter, Synaptophysin‐pHluorin, to detect synaptic vesicle fusion. At rest, because of the acidic conditions inside the lumen of the vesicle, the fluorescence is quenched. However, stimulation causes vesicular fusion to the plasma membrane that transiently equilibrates the exposed lumen to the neutral extracellular pH, leading to an increase in the observed fluorescence (Burrone et al., 2006). Hippocampal neurons were transfected with plasmids encoding shRNAs targeting CK1α or CK2α, or a scrambled shRNA, 4 days prior to the SypHy assay and neurons were monitored under resting and stimulated conditions (Figure 2a). Under basal control conditions SypHy fluorescence was low, as shown by representative images in Figure 2b. Following mild stimulation (2 s, 40 APs @ 20 Hz; (Girach et al., 2013; Tang, Craig, & Henley, 2015; Craig et al., 2015)), no differences were detected in evoked release between the CK1α, CK2α or scrambled shRNA‐expressing neurons (Figure S1a). These data suggest neither CK1α nor CK2α knockdown affect the pool of primed, docked vesicles, commonly referred to as the Readily Releasable Pool (RRP).

In contrast, more intense stimulation (45 s, 900 APs @ 20Hz; (Craig et al., 2015; Girach et al., 2013; Tang et al., 2015)) to trigger fusion of the reserve vesicle pool resulted in a dramatically greater peak of SypHy fluorescence signal in CK2α KD compared to scrambled or CK1α shRNA‐expressing neurons (Figure 2c). In addition, the peak fluorescence in CK2α knockdown cells occurred slightly later after stimulation than in control shRNA or CK1α knockdown cells (~40 s for CK2α KD versus ~36 s for control cells and ~37 s for CK1α KD cells).

To quantify the magnitude of fluoresence changes across time, the areas under the curves encompassing the peak signal (Figure 2c, red lines, 35 s and 45 s) were calculated (Figure 2d). The results indicated that significantly more vesicle fusion occurs in response to intense stimulation in the pre‐synaptic terminals of CK2α knockdown neurons than from control or CK1α KD cells.

We also analysed the rates of the fluorescense decay phase, corresponding to vesicle internalization and reacidification, by normalizing to the peak evoked signal of each (Figure 2e,f). The decay phases from 40 s to 90 s following 900 APs @ 20Hz (green line in Figure 2c) were all best described by exponential functions. There were no clear differences in the rate of signal decay (Figure 2e,f) or tau value (Figure S2a) suggesting that the increase in fluorescence in the CK2α knockdown cells is because of an enhanced vesicle exocytosis rather than decreased vesicle endocytosis/reacidification.

### 3.3 Over‐expressing CK2α reduces pre‐synaptic release

**Figure 3:**
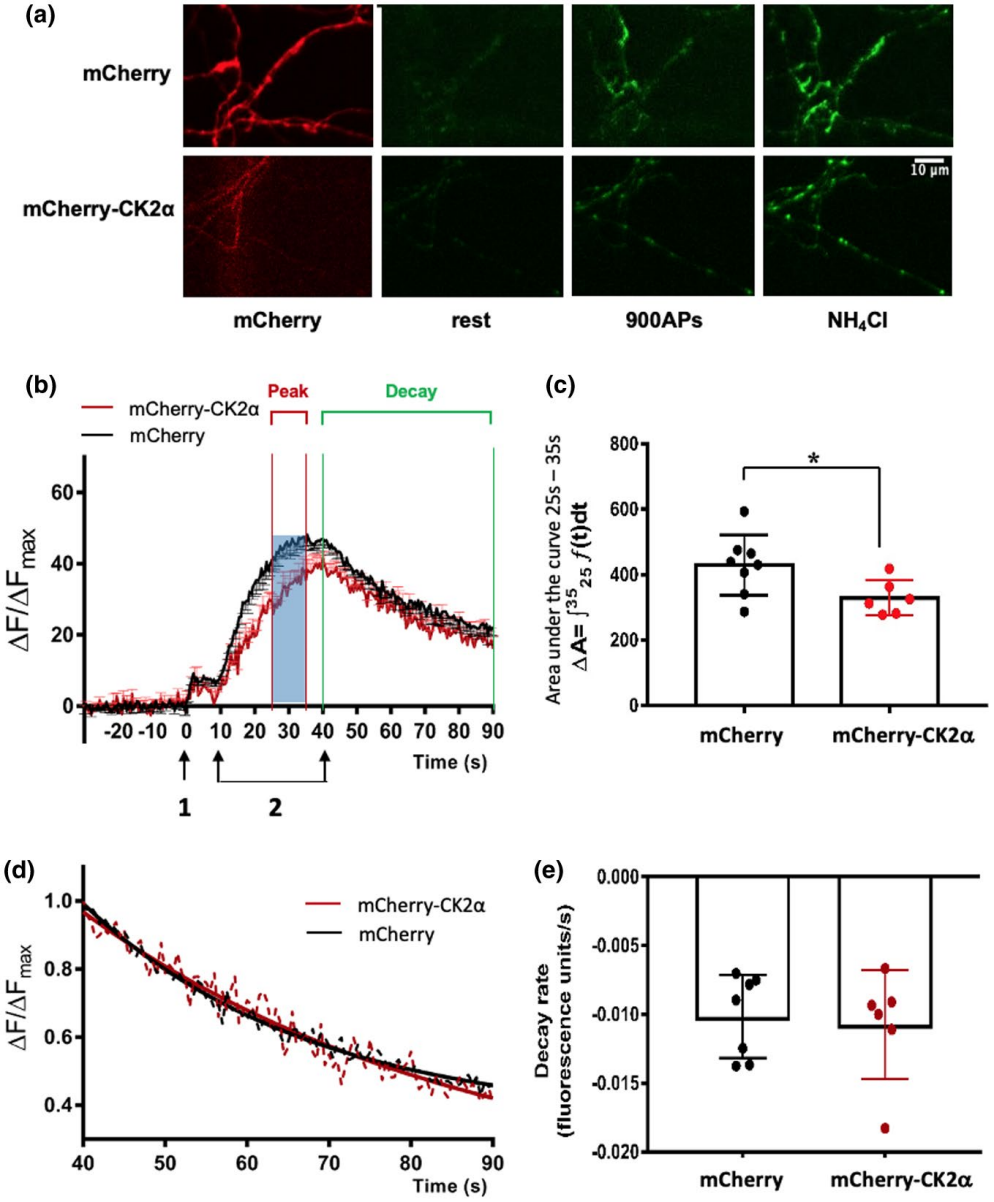
Over‐expressing Casein kinase 2α (CK2α) reduces the number synaptic vesicles released from the reserved pool in neurons. (a) Representative images of neurons transfected with synaptophysin‐pHluorin (SypHy) and either mCherry alone or mCherry‐CK2α. Illustrative images of SypHy fluorescence prior to stimulation [rest, 0 s in (a)], after a stimulation at 900 action potentials (APs) at 20Hz (900 AP; 40 s) and after the NH_4_Cl wash to reveal maximal fluorescence. Scale bar = 10µm. (b) Exocytosis profile measured by SypHy signal in control (*N* = 8 cells from at least three independent cell culture preparations) versus neurons over‐expressing mCherry‐CK2α (*N* = 6). The stimulation protocols were as in Figure 2. SypHy fluorescence was normalized to baseline and expressed as a percentage of total SypHy signal obtained by an NH_4_Cl wash. Graph shows mean ± *SEM*. (c) Quantification areas under the curves from 25 to 35 s. Areas under the curve data are presented as mean ± *SD*. *p < .05, Student's *t* test, *N* = 8 cells from at least three independent cell culture preparations for mCherry control and *N* = 6 for mCherry‐CK2α. (d) SypHy fluorescence decay profiles between 40 and 90 s were normalized to the peak evoked signal. Decay profiles are all best described by exponential functions, and are presented as mean only without error bars for clarity. (e) Quantification of data presented in (d). There was no difference in the decay profiles of control and CK2α‐over‐expressing neurons. Graph shows mean ± *SD*. *p < .05, Student's *t* test, *N* = 7 cells from at least three independent cell culture preparations for mCherry control and *N* = 6 for mCherry‐CK2α

Since CK2α knockdown increased exocytosis. we next investigated the effects of CK2α over‐expression on vesicle release. Rat hippocampal neurons were transfected with plasmids to over‐express mCherry alone or mCherry‐CK2α. SypHy release assays were performed 3 days after transfection. Representative images of the neurons are shown in Figure 3a. In neurons over‐expressing CK2α there was a slower rise in SypHy fluorescence and a pronounced difference in the areas under the curve between 25 s and 35 s following stimulation (900 APs @ 20Hz; Figure 3b), however, consistent with the results in Figure 2, there was no effect on the RRP (Figure S1b). Quantification of the data during this time period showed significantly decreased release in CK2α‐over‐expressing neurons compared to control cells (Figure 3c). Again, there were no differences in the normalized fluorescence decay profiles or tau during the internalization process (40–90 s) between control and CK2α‐expressing neurons (Figure 3d,e; Figure S2b). Together, these data are consistent with reduced exocytosis of releasable vesicles from the RP in cells over‐expressing CK2α.

### 3.4 Preventing CK2α‐mediated phosphorylation of Stx‐1a enhances pre‐synaptic vesicle exocytosis

We then tested if the effect of CK2α on synaptic vesicle release requires phosphorylation of Stx‐1a at Ser14. We knocked down endogenous Stx‐1a and replaced it with either shRNA‐insensitive wild‐type or a CK2α phosphorylation‐deficient mutant in which we substituted Asp17, which is located at the centre of the CK2α phosphorylation consensus site, with an alanine (D17A; (Rickman & Duncan, 2010)). The Munc18‐1‐open‐Syntaxin‐1a interaction involves Ser14 and is required for SNARE complex formation and fusion (Sudhof, 2014). Thus, the D17 mutant is more informative than mutation of Ser14 to investigate CK2 phosphorylation of Syntaxin‐1a as it avoids disruption of the interaction between Syntaxin‐1a and Munc18 (Rickman & Duncan, 2010). These knockdown‐replacement constructs were expressed in hippocampal neurons for 5 days, as described previously (Craig et al., 2015), prior to SypHy functional assays. Unfortunately, Stx‐1a knockdown in the absence of a rescue construct severely compromised neuronal health, meaning assessment of vesicle exocytosis upon Stx‐1a knockdown was not possible.

**Figure 4:**
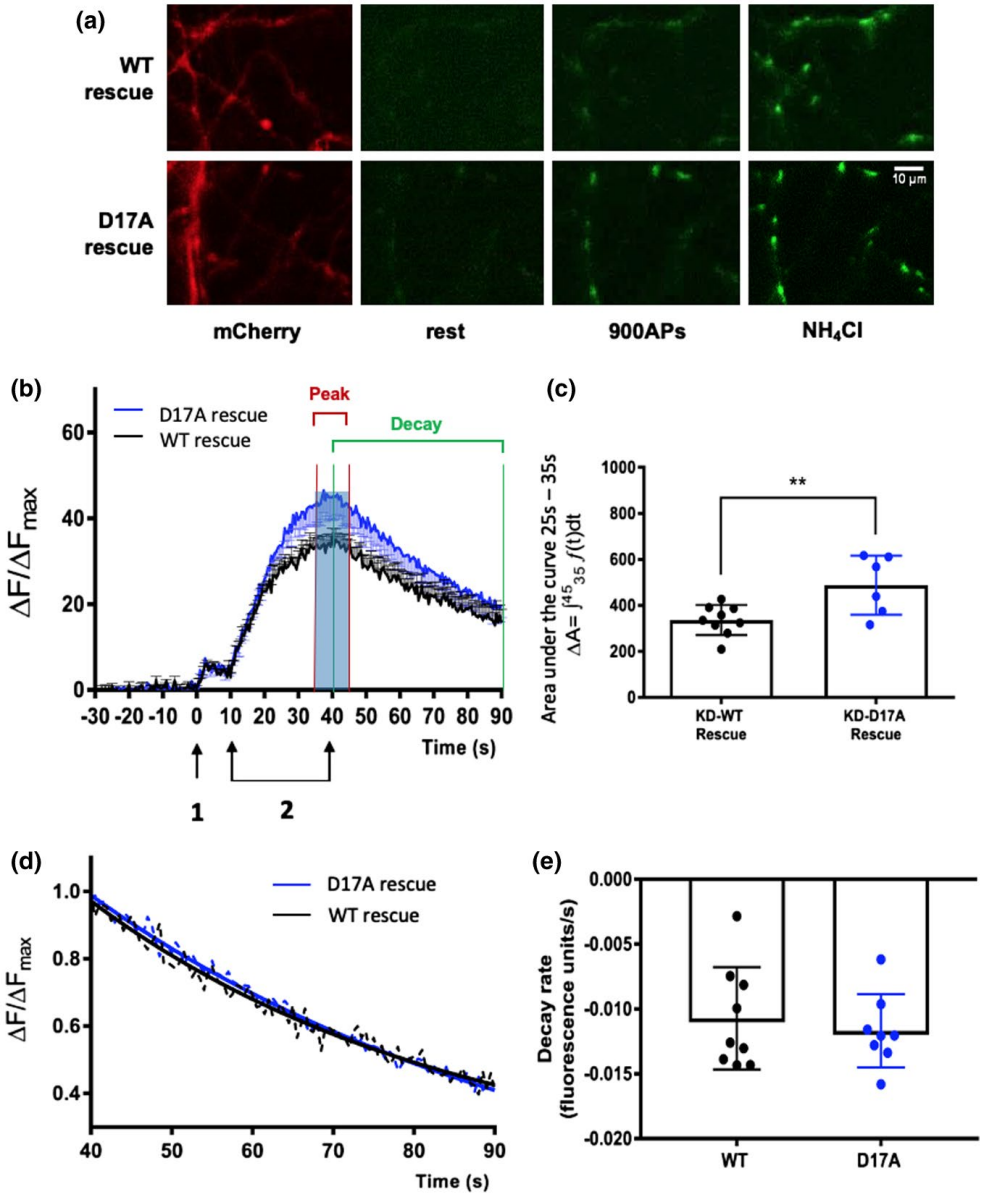
Syntaxin‐1a (Stx‐1a) phosphorylation regulates the number of synaptic vesicles released from the reserved pool. (a) Representative images of neurons transfected with synaptophysin‐pHluorin (SypHy) plus either wild‐type or D17A Syt‐1a‐IRES‐mCherry knockdown‐rescue constructs. Illustrative images of SypHy fluorescence at rest prior to stimulation, after a stimulation at 900 APs at 20Hz (900 AP; 40 s) and after the NH_4_Cl wash to reveal maximal fluorescence. Scale bar = 10µm. (b) Exocytosis profile measured by SypHy signal in Stx‐1a knockdown (KD) neurons rescued with either wild‐type (WT) Stx‐1a (*N* = 9 cells from at least three independent cell culture preparations) or Stx‐1a‐D17A mutant (*N* = 6). WT rescue group data are presented as mean + *SEM*, D17A as mean – *SEM*. (c) Quantification of peak areas under the curves from 35 to 45 s. Graph shows mean ± *SD*. **p < .01, Student's *t* test, *N* = 9 cells from at least three independent cell culture preparations for WT rescue control and *N* = 6 for Stx‐1a‐D17A rescue. (d) SypHy fluorescence decay profiles between 40 and 90 s normalized to the peak evoked signal. Decay profiles are all best described by exponential functions, and are presented as mean only without error bars for clarity. (e) There was no difference in the decay rate between Syntaxin‐1a KD WT‐rescue, D17A rescue. Graph shows mean ± S.D. *p* > .05, Student's *t* test, *N* = 9 cells from at least three independent cell culture preparations for WT rescue control and *N* = 8 for Stx‐1a‐D17A rescue

As shown in the representative images (Figure 4a) and the fluorescence profiles (Figure 4b), 900AP stimuation elicited significantly greater SypHy fluorescence signals in the non‐phosphorylatable D17A mutant compared to the wild‐type Stx‐1a‐expressing neurons, which were comparable to the CK2 knockdown cells. Again, no effect was seen on mild (40AP) stimuation, indicating that these effects were specific to the RP, and did not effect RRP size or the kinetics of its fusion (Figure S1c). Analysis of the areas under the curves encompassing the peak signal (35–45 s) showed that significantly more vesicles were released in the non‐phosphorylatable Stx‐1a‐expressing cells than from cells rescued with wild‐type Stx‐1a (Figure 4c). The rates of signal decay and tau (endocytosis) were similar between all groups (Figure 4d,e; Figure S2c), again indicating the difference in fluorescence signals were predominantly caused by increased exocytosis of reserve pool vesicles in the D17A rescue cells rather than reduced endocytosis of vesicles during the endocytosis phase.

## 4 DISCUSSION

Here, we show that pre‐synaptic vesicle release is enhanced by knockdown of CK2α. Our data demonstrate that Stx‐1a is phosphorylated by CK2α, and not by CK1α, at residue Ser14 in cultured neurons. Interestingly, in our experiments no apparent differences were detected in the release profiles of primed/rapidly releasable vesicles of the RPP among neurons expressing CK1α shRNA, CK2α shRNA or a scrambled version of shRNA, suggesting that partial loss CK2α function does not impede priming or fusion of docked vesicles. However, the peak evoked fluorescence signal from CK2α KD cells was significantly higher than that from control cells during stimulation at 900APs at 20Hz. We interpret these data as an indication of an increase in the total number of releasable synaptic vesicles, possibly reflecting an increase in the reserve pool. Moreover, the 900 APs at 20Hz invoked release in CK2α KD cells continued to increase until the stimulation was stopped, whereas in control and CK1α knockdown neurons the release peaked and began to drop during the stimulation period, reaching a peak release at ~35.5 s compared to CK2α KD cells at ~40 s. Consistent with a model whereby CK2α‐mediated phosphorylation of Stx‐1a limits the availability of synaptic vesicles for fusion, over‐expression of CK2α produced an opposite effect in the total number of releasable synaptic vesicles compared to that observed in CK2α KD cells, although this effect was less pronounced.

It should be noted that since we did not include bafilomycin in our exocytosis experiments to prevent quenching of signal from endocytosed SypHy reporter, our exocytosis profiles represent ‘net’ exocytosis under the conditions used. However, since the fluorescence signal decay profiles, which represent endocytosis and reacidification of vesicles, were unaltered by manipulation of CK2α, we attribute the observed changes in exocytosis profile to represent changes in vesicle exocytosis, as opposed to altered endocytosis.

Taken together, the data suggest that loss of CK2α function disrupts control of the total number of synaptic vesicles available for release without affected the fusion kinetics or size of the RRP. This is consistent with a previous report that demonstrated the kinase activity of CK2α and its interaction with CK2β is involved in synapse integrity in *Drosophila* motor neurons (Bulat et al., 2014).

CK2α phosphorylates Stx‐1a at Ser14 and the consensus sequence for CK2α binding is highly evolutionarily conserved in Stx‐1 orthologs (Rickman & Duncan, 2010). Preventing phosphorylation at this site by knockdown of Stx‐1a and replacement with a mutant that cannot be phosphorylated by CK2α (Stx‐1a D17A) did not alter the release of vesicles in response to short electrical stimulation, suggesting that CK2α phosphorylation of Stx‐1a does not affect vesicles already docked and primed at the pre‐synaptic terminal. It has been reported that Ser14 phosphorylation inhibits transition of the Stx1A‐Munc18 interaction from mode 1 to mode 2 (Rickman & Duncan, 2010). As this transition would be predicted to have already occurred in the docked/primed vesicle of the RRP, increasing this phosphorylation would be predicted to slow down exocytosis specifically of the RP, consistent with our data.

Moreover, replacement of Stx‐1a with the CK2α‐insensitive D17A mutant enhanced total vesicle release in response to sustained stimulation, again in support of our data using knockdown/over‐expression of CK2α. Thus, our data indicating that inhibiting Stx‐1a phosphorylation (either by CK2α KD or expression of Stx‐1a D17A) does not increase the exocytosis rate, but increases the total amount of vesicle fusion suggests that phosphorylation of Ser14 of Stx1a by CK2α plays a critical role in limiting the total number of releasable synaptic vesicles and diminishing the extent of neurotransmitter release during intense activation.

This mechanism may exist to limit pre‐synaptic rundown in response to high intensity firing of specific neuronal pathways, or to limit release of excitatory neurotransmitter to prevent excitotoxicity or epileptiform activity. Indeed, consistent with this hypothesis, several mutations in CK2a have recently been identified which lead to intellectual disability and epilepsy syndromes (Nakashima et al., 2019).

Previous work has demonstrated that pharmacological blockade of CK2α led to a reduction of Stx‐1a phosphorylation at Ser14, and an enhancement of glutamate release from synaptosomes (Gil et al., 2011). Our results agree with and extend these observations by providing evidence for a specific role of CK2α‐mediated phosphorylation of Stx‐1a in controlling exocytosis from the reserve pool of synaptic vesicles in intact neurons.

In conclusion, our data indicate that CK2α plays an important role in the regulation of neurotransmitter release. While release of docked/primed vesicles was insensitive to alterations in CK2α levels, sustained vesicle release was bidirectionally modulated by altering CK2α levels, with knockdown of CK2α enhancing the apparent total number of releasable synaptic vesicles in response to sustained stimulus. Furthermore, this effect was mimicked by specifically blocking CK2α‐mediated phosphorylation of Syntaxin‐1a at Ser14. Taken together, our data demonstrate that CK2α limits the availability of releasable synaptic vesicles, primarily through phosphorylation of Stx‐1a.

## CONFLICT OF INTEREST

The author(s) declare no competing interests.

## Material availability statement

Custom‐made materials will be shared upon reasonable request upon contacting the corresponding author.

## Supporting information

Fig S1‐S3

## Data Availability

All data generated or analysed during this study are included in this published article.
