# Supplementary material for: Phosphorylation of Syntaxin‐1a by casein kinase 2α regulates pre‐synaptic vesicle exocytosis from the reserve pool
Source: J Neurochem. 2020 Sep 6;156(5):614–23. doi: 10.1111/jnc.15161 (PMC8237229; doi:10.1111/jnc.15161)
Supplement: Supplementary file 1 — Fig S1‐S3 [file JNC-156-614-s001.pdf]

Supplement File

Phosphorylation of Syntaxin-1a by casein kinase 2α (CK2α) regulates presynaptic vesicle exocytosis from the reserve pool.

Vanilla (Hua) Shi, Tim J. Craig[#], Paul Bishop, Yasuko Nakamura,
Dan Rocca, Kevin A. Wilkinson, and Jeremy M. Henley*

School of Biochemistry, Centre for Synaptic Plasticity, Biomedical Sciences Building,
University of Bristol, Bristol, BS8 1TD, UK

[#] Department of Applied Sciences, University of the West of England,
Coldharbour Lane, Bristol, BS16 1QY, UK

*please address correspondence to j.m.henley@bristol.ac.uk (Jeremy Henley)

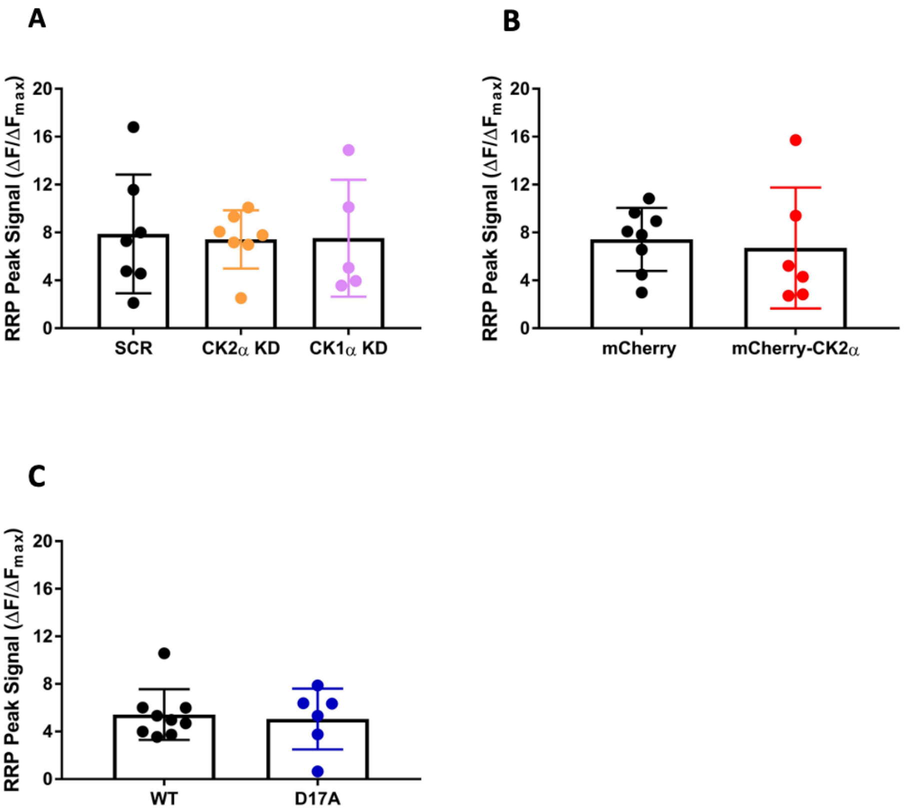

Supplementary Figure 1 Quantification of peak RRP signals from data shown in Figures 2-4

(A) Quantification of the peak RRP signal from data shown in Figure 2C. One-way ANOVA followed by Tukey's *post hoc* multi-comparison, no significant differences between groups. Data are presented as mean ± SD. SCR N=7 cells from at least 3 independent cell culture preparations; CK1α KD N=5; CK2α KD N=7.

(B) Quantification of the peak RRP signal from data shown in Figure 3B. Student t-test, no significant difference between groups. Data are presented as mean ± SD. mCherry N=8 cells from at least 3 independent cell culture preparations; mCherry-CK2α N=6.

(C) Quantification of the peak RRP signal from data shown in Figure 4B. Student t-test, no significant difference between groups. Data are presented as mean ± SD. WT rescue N=9 cells from at least 3 independent cell culture preparations; D17A rescue N=6.

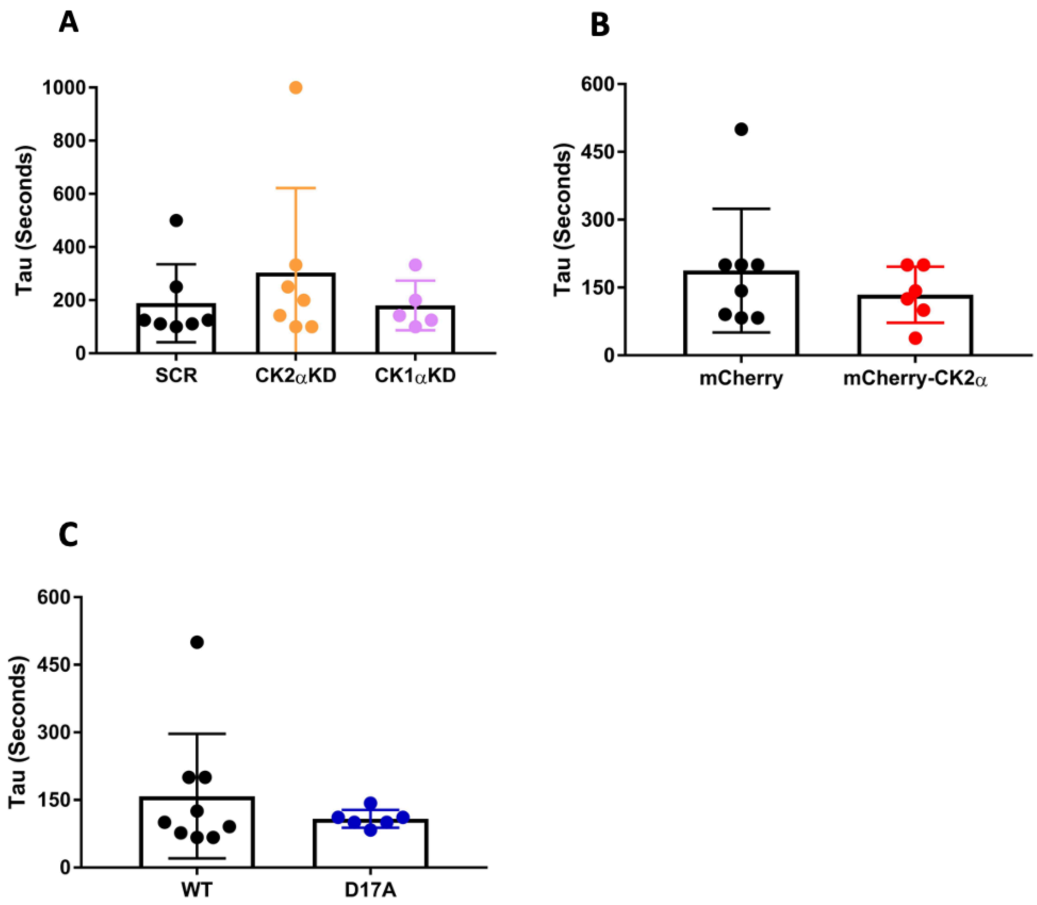

Supplementary Figure 2 Analysis of tau values for the fluorescence decay profiles shown in Figures 2-4

(A) Quantification of the tau values for the fluorescence decay shown in Figure 2F. Kruskal-Wallis followed by Dunn's multiple comparisons test, no significant differences between groups. Data are presented as mean ± SD. SCR N=7 cells from at least 3 independent cell culture preparations; CK1α KD N=5; CK2α KD N=7.

(B) Quantification of the tau values for the fluorescence decay shown in Figure 3E. Mann-Whitney test, no significant difference between groups. Data are presented as mean ± SD. mCherry N=8 cells from at least 3 independent cell culture preparations; mCherry-CK2α N=6.

(C) Quantification of the tau values for the fluorescence decay shown in Figure 4E. Mann-Whitney test, no significant difference between groups. Data are presented as mean ± SD. WT rescue N=9 cells from at least 3 independent cell culture preparations; D17A rescue N=6.

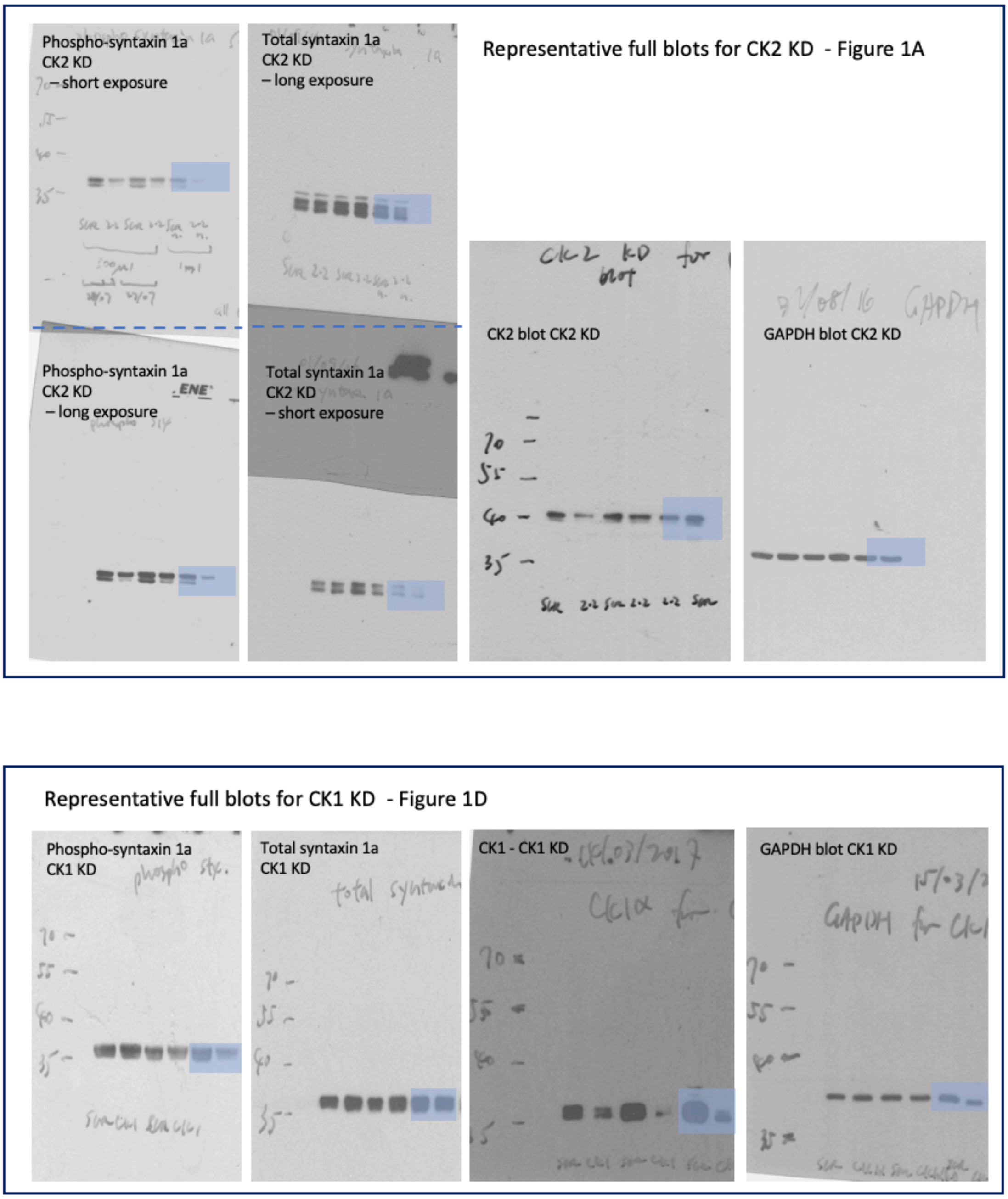

Supplementary Figure 3 **Representative full blot raw data of Western blots shown in Figure 1**
